# Comprehensive Analysis of Immune-Related Prognosis of TK1 in Hepatocellular Carcinoma

**DOI:** 10.3389/fonc.2021.786873

**Published:** 2022-01-21

**Authors:** Qun Cai, Mingyan Zhu, Jinnan Duan, Hao Wang, Jingdan Chen, Yixin Xiao, Yangqin Wang, Jianfang Wang, Xuewen Yu, Hui Yang

**Affiliations:** ^1^ Department of Infectious Diseases and Liver Diseases, Ningbo Medical Center Lihuili Hospital, Affiliated Lihuili Hospital of Ningbo University, Ningbo, China; ^2^ Department of Infectious Diseases, Shaoxing People’s Hospital, Shaoxing, China; ^3^ Department of Infectious Diseases, State Key Laboratory for Diagnosis and Treatment of Infectious Diseases, National Clinical Research Center for Infectious Diseases, Collaborative Innovation Center for Diagnosis and Treatment of Infectious Diseases, The First Affiliated Hospital, College of Medicine, Zhejiang University, Hangzhou, China

**Keywords:** liver hepatocellular carcinoma, thymidine kinase 1, biomarker, overall survival, signature, nomogram

## Abstract

Increased expression of TK1 is associated with the progression of a variety of tumors. However, the relationship of TK1 expression with immune cell infiltration and its prognostic value in hepatocellular carcinoma (HCC) are still unknown. In this study the TCGA database was used to evaluate TK1 expression and its impact on survival in patients with HCC. Compared with normal tissue, TK1 in the liver tissue of patients with HCC was significantly up-regulated at both the mRNA and protein levels. Furthermore, TK1 expression was significantly related to pathological stage, tumor stage and lymph node metastasis, with high TK1 expression being an unfavorable prognostic factor for HCC. TK1 expression was also significantly associated with the infiltration of B cells, T cells, and dendritic cells in HCC. Single-cell sequencing analysis revealed that TK1 was associated with relatively large changes in T cells, especially gamma-delta T cells. A prognostic risk score based on TK1-related immune genes (CD40LG and TNFRSF4) was established using COX regression analysis. By integrating the immune-related risk score model with clinical features, a nomogram was constructed to predict the survival rate of HCC patients (1 year, 3-year and 5-year AUC of 0.782, 0.783 and 0.771, respectively). Knockdown of the target gene for TK1 was found to have significant anti-apoptosis and pro-proliferation effects on HepG2 cells. The level of TK1 in the serum and liver tissue of patients with HCC was significantly increased relative to healthy controls. These findings highlight the role of TK1 in the tumor immune response of HCC patients and in the proliferation and apoptosis of HepG2 cells. TK1 could therefore be a potential immunotherapy target for HCC patients, while the two immune genes related to TK1 (CD40LG And TNFRSF4) may be promising prognostic biomarkers in HCC.

## Introduction

Hepatocellular carcinoma (HCC) is the most common type of liver cancer and is characterized by an extremely high recurrence rate and heterogeneity (1). Chronic viral infection with hepatitis B (HBV) or C (HCV) is the main cause of HCC (2), and in recent years the incidence and mortality of this disease have gradually increased (3). Treatment for HCC varies according to the stage of disease. Surgical resection or local ablation is generally used for early stage HCC, trans arterial chemoembolization (TACE) is used for intermediate or systemic HCC, while advanced HCC is mostly treated with drugs. However, HCC often develops to an advanced stage before being diagnosed, and the tumor recurrence rate is as high as 50% within 3 years after treatment (4). Although many studies have suggested there are significant changes in the expression of immune cells in HCC, the key genes that connect immunity and HCC are poorly understood.

Thymidine kinase 1 (TK1) participates in cell proliferation through the DNA salvage pathway (5) and up-regulation of TK1 is an early sign of cancer development (6). The serum level of TK1 has been found to correlate significantly with cancer stage. TK1 is therefore a potential biomarker for cancer recurrence and treatment monitoring, and may also have advantages over current biomarkers (7). Up-regulation of TK1 can promote angiogenesis and invasion by lung cancer cells, thereby accelerating tumor progression (8). TK1 has good sensitivity and specificity for the diagnosis of HCC as opposed to benign liver disease and healthy controls (9). Chronic liver inflammation plays a key role in the development of HCC and hence use of the immune response as a potential treatment for HCC is currently very topical. Based on a comprehensive analysis of TK1-related immune prognosis in HCC, the aim of this study was to better understand the potential role of TK1 in the pathogenesis of HCC, as well as its potential as a tumor biomarker and therapeutic target.

## Materials and Methods

### Data Collection

mRNA sequencing data (FPKM normalized, level three data) and related clinical information (age, gender, survival time, TMN stage grade, etc.) of HCC patients were obtained from the TCGA database (https://portal.gdc.cancer.gov/, accessed on January 25, 2021). The mRNA sequencing data was from 374 HCC cases and 50 paracancerous cases. Since the TCGA database information is publicly available, this study did not require ethics approval or informed consent.

### Analysis of Differential TK1 Expression

Differential expression of TK1 mRNA expression between 50 paired HCC cases and adjacent normal tissues in the TCGA data was analyzed by R. Based on the median value of TK1 gene expression in TCGA-LIHC patients, 365 patients were divided into high TK1 and low TK1 expression groups. Next, the R “limma” package was used to screen for differentially expressed genes between the two groups. The screening criteria were: absolute value of log2FC > 1, and P value < 0.001. When log2FC was > 1 and P < 0.001, the gene was considered to be positively correlated to TK1. When log2FC was<-1 and P<0.001, the gene was considered to be negatively correlated to TK1. HCC patients were divided into TK1 high- and low-expression groups according to the median value of TK1 mRNA expression. The R “survival” package was used to compare overall survival (OS) between the two groups.

### Functional Enrichment Analysis of Genes

HCC tissue samples from TCGA-LIHC (n=374) were also divided into high- and low-expression groups according to the median value of TK1 mRNA expression (the absolute value of the correlation coefficient logFC>1, P<0.001). The “ggplot2” and “cluster Profiler” packages were used to perform GO and KEGG enrichment analysis in order to explore the biological functions of TK1-related genes. Furthermore, GSEA was used to explore potential pathways for the top most enriched genes in the TK1 high- and TK1-low expression groups. The parameters selected were as follows: (h.all.v7.4.symbols.gmt) the number of gene set replacements per analysis was 1000, the filter criterion NES≥1, P ≤ 0.05 and FDR≥0.25.

### The Correlation Between TK1 Expression and Immune Infiltrating Cells

The proportion of 22 immune infiltrating cell types in each sample of the TCGA-LIHC cohort was first analyzed in CIBERSORT (https://cibersortx.stanford) (10). An empirical P-value for the deconvolution of each case was then calculated after excluding samples with P≥0.05. Sixty HCC samples and normal samples were included in this analysis. We then screened 22 immune infiltrating cell types related to HCC immune infiltrating cells and TK1 expression. We also used the Tumor Immunity Estimation Resource (TIMER- https://cistrome.shinyapps.io/timer) (11) to analyze the correlation between TK1 expression and 6 immune infiltrating cell types (B cells, CD4+ T cells, CD8+ T cells, neutrophils, macrophages and dendritic cells). The purity-corrected partial Spearman’s correlation coefficient was used to evaluate the relationship between TK1 expression and immune infiltration.

### Single Cell Sequencing Data Analysis

The Seurat package was used to generate objects from HCC’s integrated single-cell sequencing data and to remove low-quality cells (12). The standard of pretreatment is that each cell expresses at least 200 genes, each gene is expressed in at least 3 cells, the mitochondrial gene content is less than 5%, and the number of QC genes is between 200-2500. In addition, the linear dimensionality reduction method was used to perform preliminary dimensionality reduction (PCA) of the data and then to cluster the data with a resolution of one. The Uniform Manifold Approximation and Projection (UMAP) (13) algorithm was then used to visualize the clustering results. The Single R package marker gene was used to annotate the cluster subtypes. Finally, the differential expression of TK1 was calculated for each cluster subtype.

### Acquisition of Immune Regulatory Genes and Construction of Prognostic Models

Immune regulatory genes related to the expression of TK1 were obtained from the TISIDB database (http://cis.hku.hk/TISIDB/)) (14). In order to prevent gene co-expression from affecting the prediction model, log2(TPM+1) was used to normalize the TPM value. First, univariate Cox analysis was used to evaluate the relationship between these immune regulatory genes and the prognosis of HCC patients. The differential immune regulatory genes were selected for multivariate Cox analysis to construct an HCC prognostic model of immune genes related to TK1. The riskScore is calculated using the following formula:


$$\text{riskScore}=\sum \beta_{\text{i}}*{\text{Exp}}_{\text{i}}$$

β_i_ is the coefficient of each included gene, while Exp_i_ is the expression level of each included gene. TCGA-LIHC patients were divided into high- and low-risk groups according to the median value of the riskScore. The R “survival” package was then used to analyze the difference in OS between these two groups.

### Construction and Verification of an HCC Clinical Nomogram

The clinical information in the TCGA-LIHC cohort was integrated by inserting riskScore, age (<=65 and >65 years), M (M0 and M1), N (N0 and N1), T (T1+T2 and T3+T4), and stage (stage I+II and stage III+IV). Univariate and multivariate Cox regression analysis were used to identify significant clinical variables. These variables and the riskScore were used in the R “rms” package to construct a nomogram to predict the prognosis of HCC patients. Meanwhile, the calibrated ROC curve and decision curve analysis were used to estimate the prognostic value of nomograms at 1, 3 and 5 years follow-up (15, 16). A calibration curve was constructed using the bootstrap method (1000 cycles) to show the deviation between the predicted value and the actual probability of occurrence.

### Validation of TK1 by *In Vivo* and *In Vitro* Experiments

We recruited 100 HCC patients and 100 healthy controls in order to compare the expression of TK1 and AFP in the serum of these two groups. Liver tissue sections from both groups were also obtained to explore the differential expression of TK1 in liver tissues. TK1 in the liver cancer cell line HepG2 was knocked down to study the effect on the growth cycle of these cells. Lipopolysaccharide (LPS: 1000ng/ml) was used to induce the inflammatory response in HepG2 cells. After 24 hours of LPS stimulation, cells were collected for the evaluation of apoptosis by flow cytometry and cell proliferation by CCK-8.

### Validation of RiskScore in ICGC

RNA sequencing and clinical data for 240 cases of primary HCC were downloaded from the International Cancer Genome Consortium (ICGC, https://dcc.icgc.org) website for use as a validation cohort (ICGC-LIRI-JP). These samples are mainly from Japanese patients with liver cancer caused by HBV or HCV infection. The risk value for each patient in this ICGC-LIRI-JP cohort was calculated according to the risk formula. The validation cohort was divided into high- and low-risk groups by using the median value of the TCGA-LIHC cohort risk score as a cut-off. Six cases with missing information or who were lost to follow-up were excluded, giving a total of 234 HCC patients included in the validation study.

### Statistical Analysis

R 4.0.1 and SPSS software (v23, Chicago, IL) were used for data analysis and interpretation. The survival package was used for Cox regression analysis and Kaplan-Meier curves, the nomogram was generated using the R “rms” package, and all visualizations were obtained using the ggplot2 package. Categorical data was used for the chi-square test, continuous variables were expressed as frequency, percentage, or mean/standard deviation. Normally distributed variables were analyzed using the t test and non-normally distributed variables using the Mann-Whitney test. *P ≤* 0.05 was set as representing a statistically significant difference.

## Results

### Differential Expression and Prognosis of TK1

**Figure 1:**
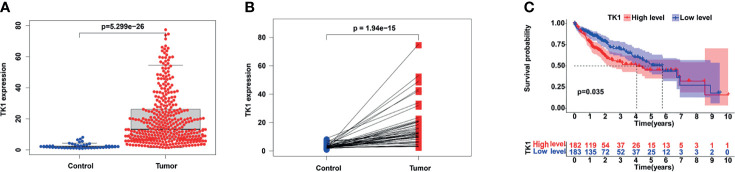
Expression of TK1 in hepatocellular carcinoma (HCC) and its prognostic value. **(A)** Differential TK1 expression in tumor tissue and normal tissue. **(B)** Differential expression of TK1 in paired liver cancer and adjacent tissues (N=50). **(C)** Kaplan–Meier survival curves according to TK1 expression and based on the TCGA-LIHC dataset (group cutoff=median).

The expression of TK1 mRNA in HCC tumor tissues was significantly higher than in normal tissues (**Figure 1A**). A paired comparison in 50 HCC patients showed that TK1 mRNA expression in cancer tissue was significantly higher than in adjacent normal tissue (**Figure 1B**). HCC patients were divided into high- and low-expression groups based on the median TK1 value. HCC patients in the TK1 high-expression group had poorer prognosis (**Figure 1C**).

### Functional Enrichment of TK1 Related Genes

**Figure 2:**
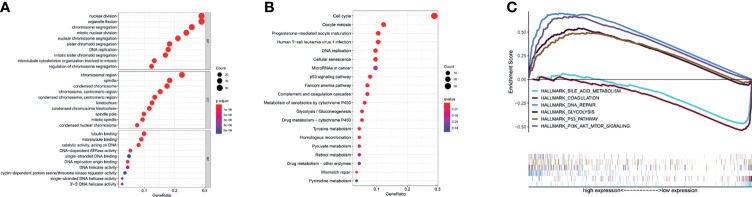
Functional enrichment of TK1-related genes (TK1-RGs). **(A)** GO plots of the TK1-RGs. **(B)** KEGG plots of the TK1-RGs. **(C)** GSEA showed that TK1 is involved in DNA repair, the P53 pathway, PI3K AKT MTOR signaling, Glycolysis, Coagulation, and bile acid metabolism.

We next examined genes that are significantly related to TK1. Gene Ontology (GO) analysis showed these genes are mainly involved in: “nuclear division”, “organelle fission”, “DNA replication”, and “mitotic nuclear division” in Biological Process (BP); “chromosomal region” and “condensed chromosome” in Cellular Component (CC); “tubulin binding”, “microtubule binding”, “catalytic activity acting on DNA”, and “single−stranded DNA binding” in Molecular Function (MF) (**Figure 2A**). Kyoto Encyclopedia of Genes and Genomes (KEGG) analysis suggests these genes are mainly involved in the regulation of pathways such as “cell cycle”, “oocyte meiosis”, “human T−cell leukemia virus 1 infection”, “DNA replication”, “microRNAs in cancer” and “p53 signaling pathway” (**Figure 2B**). Further investigation by Gene Set Enrichment Analysis (GSEA) found the “DNA repair”, “P53 pathway”, “PI3K AKT MTOR signalling” and “glycolysis” pathways were significantly up-regulated when TK1 was highly expressed, while the “coagulation” and “bile acid pathways” pathways were significantly down-regulated (**Figures 2C** **–H**). These results indicate that TK1 may play an important role in tumor growth and metabolism.

### Differential Expression of Tumor Immune Infiltrating Cells and Their Correlation With TK1 Expression

**Figure 3:**
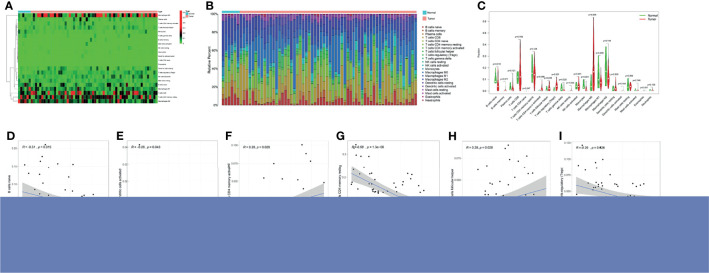
Immune cell infiltration and TK1 expression in HCC. **(A)** Heatmap of immune cell expression in different tissue samples (normal vs tumor). **(B)** Histogram of the relative expression of immune cells in different tissue samples (normal vs tumor). **(C)** Violin chart showing differences in immune cell expression in different tissue samples (normal vs tumor). Correlation between TK1 expression and six immune cell infiltrates **(D–I)** cells CD4 naive (P=0.018), Tregs (P=0.001), Macrophages M0 (P=0.006) were all significantly higher in the HCC group. In contrast, the proportion of B cells naïve (P=0.047), T cells gamma delta (P=0.02), monocytes (P=0.001) was lower.

A differential analysis of immune infiltrating cells between HCC and normal liver tissue controls was performed. “T cells CD4 naive”, “T cells regulatory (Tregs)” and “Macrophages M0” were significantly up-regulated in HCC liver tissue, whereas “B cells naïve”, “T cells gamma delta” and “Monocytes” were significantly down-regulated (**Figures 3A** **–C**). The immune infiltrating cell types that were significantly associated with TK1 expression were “B cells naïve”, “dendritic cells activated”, “T cells CD4 memory activated”, “T cells CD4 memory resting”, “T cells follicular helper” and “T cells regulatory (Tregs)”. T cells CD4 memory resting showed a correlation coefficient of -0.58 with TK1 expression (**Figures 3D** **–I**). A summary of the immune genes that were significantly associated with TK1 expression is shown in **Supplementary Table 1**.

### Single-Cell Sequencing of TK1 in HCC

**Figure 4:**
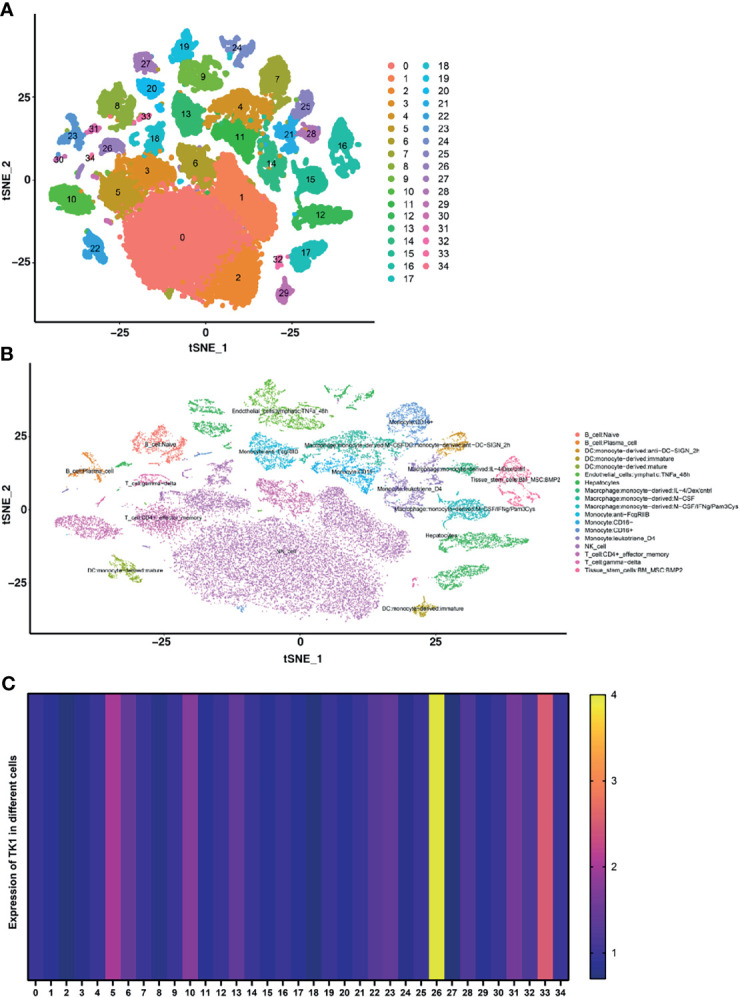
Single-cell RNA sequencing was used to identify differentially expressed genes (DEGs) in immune and non-immune cells. **(A)** The UMAP algorithm classified all DEGs into 35 clusters. **(B)** The Singler package annotates cell types for all cell subpopulations, giving a total of 18 cell types. **(C)** Heatmap for the expression of TK1 in different cells.

When HCC single-cell data was downloaded from the GEO database, 35 clustering results could be visualized (Clustering resolution=1) using the JUP.UMAP algorithm, with clear separation between the subgroups (**Figure 4A**). In order to improve annotation of the clustering results in scRNA-seq, the R package “SingleR” was applied and the 35 clustering results were annotated as 18 cell populations (**Figure 4B**). The expression of TK1 was highest in the T-cell: gamma-delta cluster (**Figure 4C**).

### Construction of a TK1-Related Immune Score and Prognosis Nomogram

**Figure 5:**
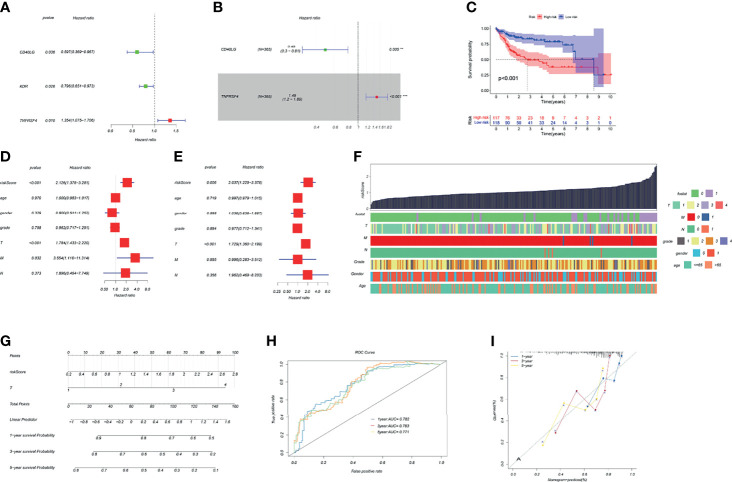
Construction of a TK1-related immune score and prognosis nomogram. **(A, B)** Univariate and multivariate Cox regression analysis of immune genes related to TK1 was used to construct the riskScore. **(C)** Kaplan–Meier survival curves for riskScore based on the TCGA-LIHC dataset (group cutoff=median). **(D, E)** Univariate and multivariate Cox regression analysis was used to identify clinical characteristics for the construction of a nomogram. **(F)** Relationship between riskScore and clinical characteristics. **(G)** Nomogram to predict prognostic probabilities in the TCGA-LIHC dataset. **(H)** Nomogram for predicting the 1-, 3-, and 5-year overall survival (OS) rates of HCC patients. **(I)** Calibration curves for 1-, 3-, and 5-year OS of HCC patients.

The HCC riskScore of immune genes related to TK1 was constructed using univariate and multivariate Cox analysis, where the riskScore = (Expression level of CD40LG*(0.49) + (Expression level of TNFRSF4*1.49)) (**Figures 5A, B**). We then divided HCC patients into high- and low-risk groups in order to assess their prognosis based on the median riskScore. The prognosis of patients in the high-risk group was poor (**Figure 5C**). Validation of the riskScore in the ICGC-LIRI-JP cohort is shown in **Supplementary Figure 1**. The area under the ROC curve was used to evaluate the performance of riskScore for predicting the 1-year and 3-year overall survival rates of TCGA-LIHC patients (**Supplementary Figure 1**). To improve the early warning prediction for HCC patients, the riskScore and clinical information were integrated. Univariate and multivariate Cox regression analysis found that riskScore and T staging have relatively superior predictive performance (**Figures 5D, E**). Further integration of the riskScore with clinical information showed that it was positively correlated with tumor stage (T stage, M stage, and N stage), with a higher riskScore correlating with worse prognosis (**Figure 5F**). The riskScore and T staging were used to construct a nomogram to predict patient survival at 1-, 3-, and 5-years follow-up. A calibration curve was drawn and the Area Under Curve (AUC) was calculated to determine the performance efficiency of the nomogram. The results for the 1-, 3-, and 5-year AUC were 0.782, 0.783 and 0.771, respectively (**Figures 5G** **–I**).

### Differences in the Clinical Expression of TK1 and Its Role in HepG2 Cells

**Figure 6:**
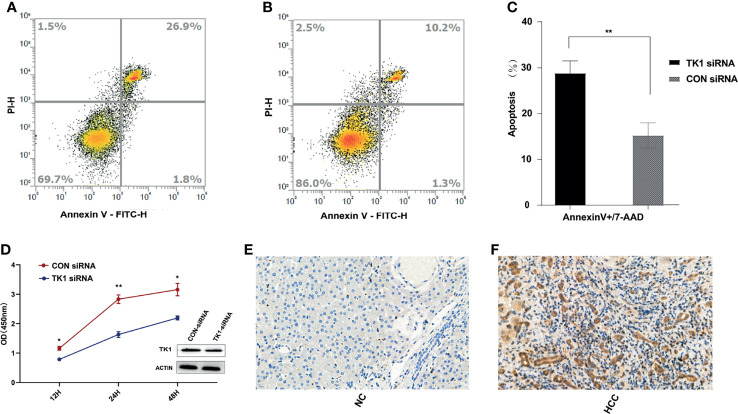
*In vivo* and *in vitro* experiments for validation of TK1. **(A)** Apoptosis assay results for the TK1-siRNA group after LPS stimulation for 24 hours. **(B)** Apoptosis assay results for the CON-siRNA group after LPS stimulation for 24 hours. **(C)** Results of triplicate experiments for the apoptosis assay in the TK1-siRNA and CON-siRNA group after LPS stimulation for 24 hours. **(D)** Cell proliferation assay results for the TK1 siRNA group and the CON-siRNA group. (All experiments used the HepG2 cell line *P < 0.05, **P < 0.01). **(E)** Expression of TK1 in the liver tissues of healthy control patients. **(F)** Expression of TK1 in the liver tissues of HCC patients.

After matching for age and gender, 100 HCC patients and 100 healthy controls were recruited in order to evaluate the serum levels of TK1 and AFP in the two groups. The TK1 and AFP serum levels in HCC patients were significantly higher than those in healthy controls (**Table 1**). Liver tissue analysis showed that the expression of TK1 in HCC patients was also significantly higher than in healthy controls (**Figures 6E, F**). *In vitro* experiments were conducted to study the effect of TK1 on the hepatocarcinoma cell line HepG2. Knockdown of TK1 expression by siRNA in the HepG2 cell line resulted in significant up-regulation of apoptosis (**Figures 6A** **–C**) and significant reduction of proliferation (**Figure 6D**) compared with the control. Therefore, these results indicate that TK1 has significant anti-apoptosis and pro-proliferation effects on HepG2 cells.

**Table 1:** Clinical characteristics of patients.

Characteristics	LIHC (n = 100)	NC (n = 100)	*P*
Age (yrs)	49.64 ± 11.80	49.40 ± 11.95	0.887
Male (%)	80% (80)	69% (69)	0.104
TK1 (pg/ml)	3.20 ± 1.76	0.91 ± 0.44	0.000
AFP (ng/ml)	246.97 ± 250.53	7.91 ± 3.90	0.000

## Discussion

The high incidence and mortality of HCC poses a serious threat to human health. Immune cells are increasingly becoming recognized as a critical element in tumor development and progression (3). Immunotherapy is also a promising new treatment method for HCC (17). Therefore, novel biomarkers and immune targets are an important area of research in HCC. Recent studies indicate that in addition to its role as a biomarker of cancer cell proliferation, TK1 is also involved in cancer cell invasion and progression (18–20), although the underlying mechanism remains unknown. In the present study, TK1 mRNA and protein levels in HCC patients were significantly up-regulated compared to healthy patients, while low expression of TK1 was found to be a good prognostic indicator. Surprisingly, enrichment analysis of TK1-related genes revealed they are involved in cell proliferation, DNA repair, the P53 pathway, PI3K AKT mTOR signaling, glycolysis and bile acid metabolism. Together, these results suggest that TK1 may play an important role in tumor growth and metabolism.

The PI3K AKT mTOR signaling pathway can inhibit cell apoptosis, promote tumor cell invasion and metastasis, and regulate angiogenesis, thus contributing to the formation of HCC (21). Glycolysis was first discovered in liver cancer and is an indicator for this disease. It is also responsible for regulating the proliferation, immune escape, invasion, metastasis, angiogenesis and drug resistance of liver cancer (22). Due to the importance of glycolysis in the progression of liver cancer, the targeting of key rate-limiting enzymes in the glycolysis pathway (eg. hexokinase 2, phosphofructokinase 1 or type M2 and pyruvate kinase) is a novel approach for HCC treatment. In addition to exerting their normal physiological functions in digestion, bile acids are also closely associated with the occurrence and development of HCC (23). PI3K AKT mTOR activation can inhibit glycolysis (24). It is well known that p53 inhibits glycolysis and gluconeogenesis *via* many downstream targets, as well as promoting mitochondrial oxidative phosphorylation to counter the Warburg effect (25). p53 is heavily involved in regulating glucose metabolism in tumor cells, including glucose transport, glycolysis and pentose phosphate pathways. The role of p53-mediated metabolic regulation in tumor suppression is related to its functions in promoting cell survival and cell death in different physiological environments (26). These results indicate that TK1 may participate in glycolysis and gluconeogenesis by activating the P53 and PI3K AKT mTOR pathways, thus promoting the development of HCC. The imbalance of bile acid metabolism and of intestinal flora involved in this imbalance, as well as abnormal expression and regulation of specific bile acid receptors are all involved in the development of HCC (27, 28). Bile acids can directly destroy the plasma membrane and cause activation of protein kinase C (PKC), p53 and nuclear factor kappa-B (NF-κB), thus inducing apoptosis and increasing inflammation. NF-κBp65 can also directly bind to FXR and inhibit its transcriptional activity, resulting in decreased expression of bile acid transporter and an increase in the level of bile acid in the liver, thus leading to inflammation of HCC (27). Taken together, these results indicate that TK1 may participate in the development of HCC through a variety of ways, and that blocking its functional sites may be a new approach for the treatment of HCC.

The present study found that Tregs were significantly up-regulated in liver cancer tissue compared with normal liver tissue, while T cells gamma delta and monocytes were significantly down-regulated. Tregs are regulatory T cells that can inhibit the function of immune cells and protect against the development of tumors. High levels of Tregs in the microenvironment of liver cancer are associated with poor prognosis (29). Some workers have suggested that tumor infiltration of T cells gamma delta is associated with better prognosis of HCC patients (30, 31). Other studies also suggest that T cells gamma delta can stimulate the production of interleukin-17A (IL-17A), thereby stimulating tumor cell proliferation, inducing angiogenesis and promoting inflammation (32–34). Single-cell sequence analysis of liver cancer tissue revealed that T cells gamma delta had the highest expression of TK1. Hence, the correlation between TK1 and T cells gamma delta in HCC and the underlying mechanism of action deserve further study. Another important finding was the correlation between TK1 and immune cell infiltration in HCC. TIMER analysis revealed a significant correlation between TK1 expression and the levels of “B cells naïve”, “activated dendritic cells”, “T cells CD4 memory activated”, “T cells CD4 memory resting”, “T cells follicular helper” and especially “Tregs”. There was a significant negative correlation of TK1 with T cells CD4 memory resting. These cells have been shown to have a positive effect on prognosis (35). TK1 expression was a factor for poor prognosis of HCC in the present study and was also significantly associated a variety of immune infiltrating cells, all of which indicates the importance of TK1 in HCC research.

We constructed an HCC riskScore of TK1-related immune genes using univariate and multivariate Cox analysis. The immune genes included in the riskScore were CD40LG and TNFRSF4. CD40LG is a co-stimulatory molecule expressed on activated CD4+ helper T cells. Decreased expression of CD40LG on the T cells of cancer patients indicates an impaired immune response. Moreover, CD40LG has a strong anti-tumor effect on HCC and other malignant tumors (36), while the CD40LG-CD40L interaction has been shown to overcome tumor-specific CD4+ and CD8+ tolerance and thus induce anti-tumor immunity (37). TNFRSF4, also known as OX40, is a co-stimulatory receptor that is expressed on T cells. It can be combined with OX40L to target the activation of NF-κB (38). Tregs are a subset of CD4+ T cells with strong immunosuppressive properties. By preventing CD8+ T cells from inhibiting the immune response and by promoting tumor escape, Tregs are often associated with poor prognosis of HCC. OX40 is a costimulatory molecule expressed by Tregs. High expression levels of OX40, as shown by immunohistochemistry and tumor genome atlas analysis, are associated with high serum alpha-fetoprotein level, vascular invasion and poor prognosis of HCC patients (39). Therapeutic antagonism of OX40 may therefore increase the response to PD-1 checkpoint inhibitors. However, little is still known about the expression of TK1 in HCC, its relationship with the clinical pathology and molecular characteristics of this disease, and its role in shaping the tumor immune microenvironment. Interestingly, TK1 expression was significantly correlated with CD40LG and OX40, and TK1 protein levels in the serum and liver tissue of HCC patients were significantly higher than in healthy controls. TK1 also showed significant anti-apoptosis and pro-proliferation effects in HepG2 cells. Therefore, further studies of TK1 may improve our understanding of HCC immunobiology and could help to break immune tolerance in HCC with poor immunogenicity, thus providing a theoretical basis for the development of anti-TK1 immunotherapy in this tumor type.

In summary, high TK1 expression is associated with poor prognosis in HCC patients and may promote the development of this cancer type by inhibiting the apoptosis of tumor cells and promoting their proliferation. TK1 is involved in the tumor immune response and may therefore be a potential target for immunotherapy of HCC. The two TK1-related immune genes CD40LG and TNFRSF4 are promising prognostic biomarkers in HCC. Finally, the prognostic nomogram constructed by integrating the riskScore of TK1-related immune genes (TNFRSF4 and CD40LG) and T staging shows good predictive performance and should prove useful for clinical treatment and medical decision-making.

## Data Availability Statement

The datasets presented in this study can be found in online repositories. The names of the repository/repositories and accession number(s) can be found in the article/**Supplementary Material**.

## Ethics Statement

The study was conducted according to the principles of the Declaration of Helsinki and approved by the ethics committee of Zhejiang University. The ethics committee waived the requirement of written informed consent for participation. Written informed consent was obtained from the individual(s) for the publication of any potentially identifiable images or data included in this article.

## Author Contributions

QC contributed to the conception and design of the review. MZ, JD, and HW wrote the manuscript. QC revised the manuscript. JC, YX, and YW collected serum and completed the follow‐up. JW, XY, and HY contributed toward the statistical analysis of this work. MZ, JD, and HW interpreted the data. All authors contributed to manuscript revision, read, and approved the submitted version.

## Conflict of Interest

The authors declare that the research was conducted in the absence of any commercial or financial relationships that could be construed as a potential conflict of interest.

## Publisher’s Note

All claims expressed in this article are solely those of the authors and do not necessarily represent those of their affiliated organizations, or those of the publisher, the editors and the reviewers. Any product that may be evaluated in this article, or claim that may be made by its manufacturer, is not guaranteed or endorsed by the publisher.

## Supplementary Material

Supplementary Figure 1 Validation of riskScore in the ICGC-LIRI-JP cohort. **(A)** Differential expression of two genes in the high- and low-risk groups. **(B)** The grade and distribution of prognostic indicators for high-risk and low-risk populations. **(C)** Comparison of survival time between high-risk and low-risk groups.

Supplementary Figure 2 RiskScore used to predict the overall survival of TCGA-LIHC patients after 1-year **(A)** and 3-year **(B)** follow-up.
