# Supplementary figures and images for: Comprehensive Analysis of Immune-Related Prognosis of TK1 in Hepatocellular Carcinoma
Source: Front Oncol. 2022 Jan 21;11:786873. doi: 10.3389/fonc.2021.786873 (PMC8814100; doi:10.3389/fonc.2021.786873)

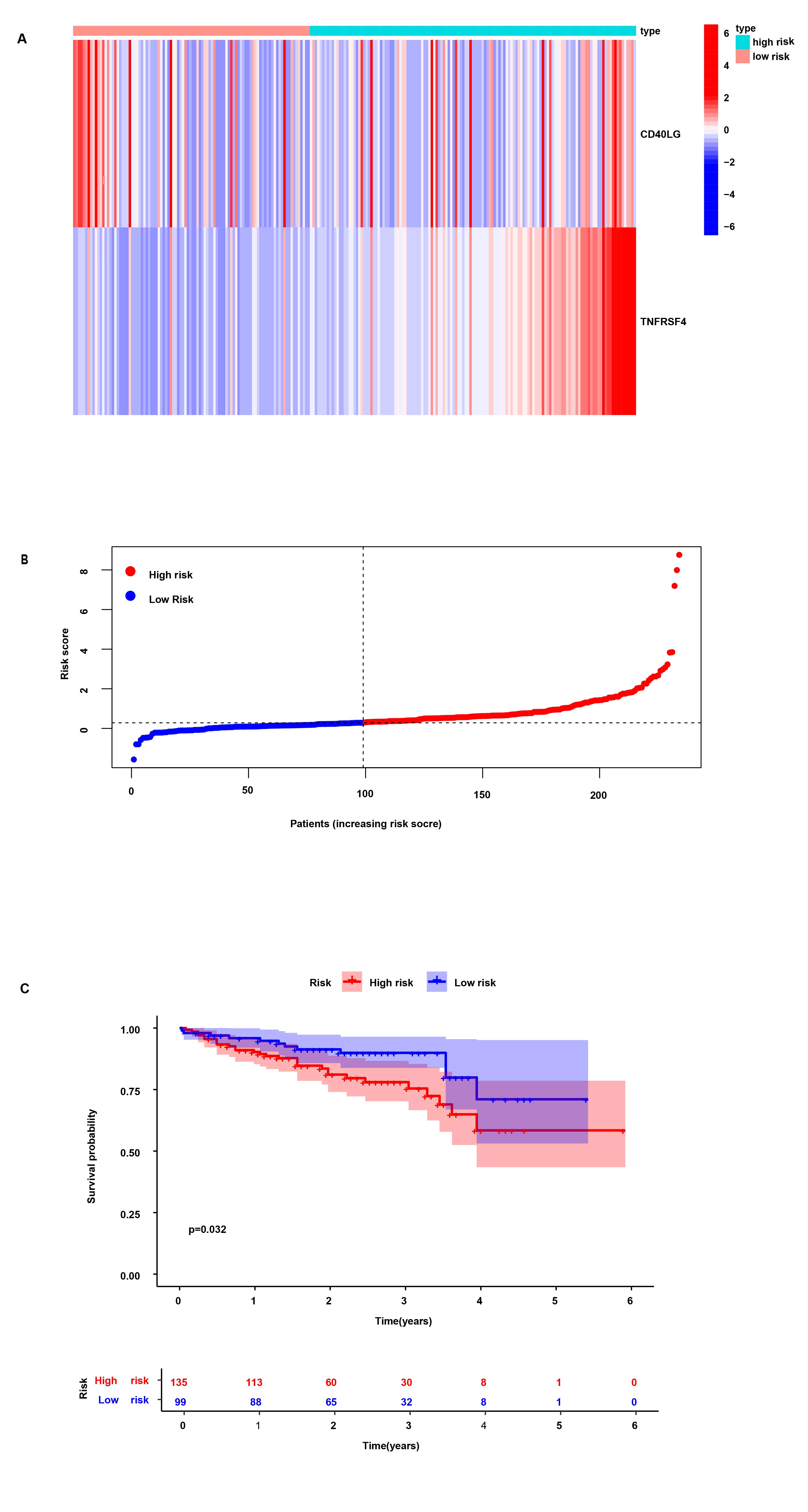

Supplement: Supplementary Figure 1 — Validation of riskScore in the ICGC-LIRI-JP cohort. (A) Differential expression of two genes in the high- and low-risk groups. (B) The grade and distribution of prognostic indicators for high-risk and low-risk populations. (C) Comparison of survival time between high-risk and low-risk groups. [file Image_1.jpg]

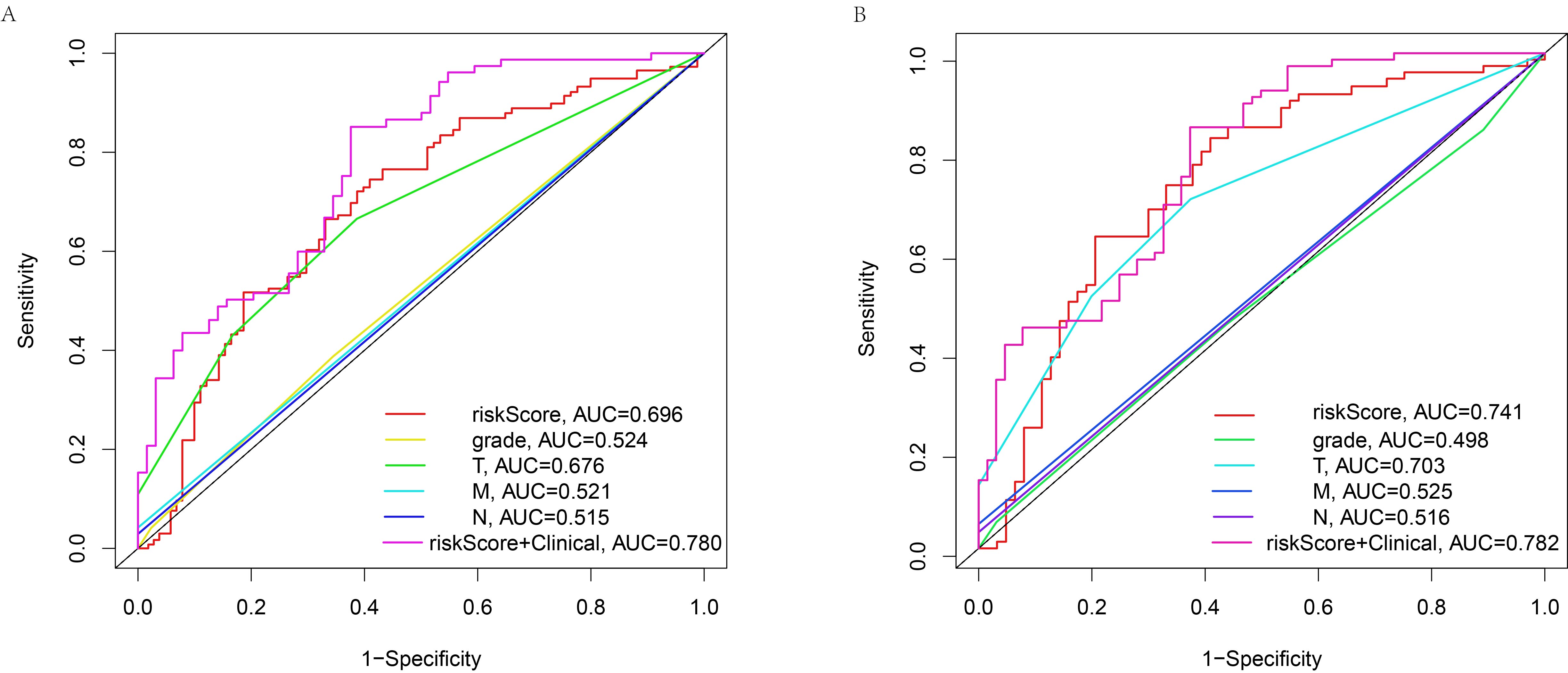

Supplement: Supplementary Figure 2 — RiskScore used to predict the overall survival of TCGA-LIHC patients after 1-year (A) and 3-year (B) follow-up. [file Image_2.jpg]
