# Supplementary material for: Comprehensive Analysis of Immune-Related Prognosis of TK1 in Hepatocellular Carcinoma
Source: Front Oncol. 2022 Jan 21;11:786873. doi: 10.3389/fonc.2021.786873 (PMC8814100; doi:10.3389/fonc.2021.786873)
Supplement: Supplementary file 3 [file Table_1.docx]

**Supplementary Table 1: Immune regulatory genes associated with TK1 in TISIDB database**

| **Related gene** | **R** | ***P*** | **Related gene** | **R** | ***P*** |
| --- | --- | --- | --- | --- | --- |
| BTLA | -0.114 | 0.028 | IL6R | -0.226 | 0.000 |
| CD96 | -0.141 | 0.006 | MICB | 0.311 | 0.000 |
| KDR | -0.485 | 0.000 | NT5E | -0.154 | 0.003 |
| LAG3 | 0.172 | 0.001 | TMEM173 | 0.322 | 0.000 |
| CD40 | 0.184 | 0.000 | TNFRSF4 | 0.190 | 0.000 |
| CD40LG | -0.249 | 0.000 | TNFRSF9 | 0.117 | 0.020 |
| CD48 | -0.127 | 0.010 | TNFRSF18 | 0.24 | 0.000 |
| CXCL12 | -0.396 | 0.000 | TNFSF9 | 0.134 | 0.009 |
| CXCR4 | -0.105 | 0.040 | TNFSF13 | -0.169 | 0.001 |
| ENTPD1 | -0.181 | 0.001 | TNFSF13B | -0.128 | 0.010 |
| HHLA2 | 0.191 | 0.000 | TNFSF14 | -0.171 | 0.001 |
| ICOSLG | -0.174 | 0.001 | TNFSF15 | -0.139 | 0.007 |
| IL6 | -0.227 | 0.000 |  |  |  |
